# Imprinted *Dlk1*-*Gtl2* cluster miRNAs are potential epigenetic regulators of lamb fur quality

**DOI:** 10.1186/s12864-023-09741-3

**Published:** 2023-10-23

**Authors:** Letian Zhang, Jiankui Wang, Ganxian Cai, Lina Ma, Zhengwei Zhao, Qing Ma, Xuemei Deng

**Affiliations:** 1 Beijing Key Laboratory for Animal Genetic Improvement & Key Laboratory of Animal Genetics, Breeding and Reproduction, Ministry of Agriculture & State Key Laboratory of Animal Biotech Breeding, China Agricultural University, Beijing, 100193 China; 2 Institute of Animal Science, Ningxia Academy of Agriculture and Forestry Sciences, 750002 Yinchuan, China

**Keywords:** *Dlk1*-*Gtl2*, Tan sheep, Hu sheep, fur, miRNA-seq, PI3K-AKT

## Abstract

**Background:**

Tan and Hu sheep are well-known local breeds in China, producing lamb fur with unique ornamental and practical values highly appreciated by consumers worldwide. Fur quality is optimal at one month of age and gradually declines with time. Despite active research on its genetic mechanism using transcriptomic and whole genome bisulfite sequencing analysis, the main effective gene locus has not been found, and its regulatory mechanism is still unclear, which limits the breeding and improvement of fur traits.

**Results:**

Scapular skin samples from newborn (1-month old) and adult (24-month old) Tan sheep were utilized for small ribonucleic acid (RNA) sequencing Principal Component Analysis (PCA) showed that the newborn and adult groups were completely separated. Differential expression analysis of micro-RNAs (miRNAs) identified 32 up-regulated miRNAs and 48 down-regulated miRNAs in the newborn groups. All up-regulated miRNAs were located in the imprinted.

*Dlk1*-*Gtl2* locus on chromosome 18, whereas all down-regulated miRNAs were distributed across the sheep chromosomes, without a clear pattern of positional consistency. Further, by systematically analyzing the target genes and signaling pathways of all 32 up-regulated miRNAs, we found that the PI3K-AKT signaling pathway has the potential to be targeted and regulated by most of the miRNAs in the *Dlk1-Gtl2 *region. In addition, we also re-analyzed miRNA sequencing data from public databases on Hu lambs (full sibling Hu lambs with high- and low-quality fur characteristics). Again, it was found that most of the up-regulated miRNAs in lambs with high-quality fur were also located in the *Dlk1*-*Gtl2* region, whereas this patter was not present for down-regulated miRNAs.

**Conclusion:**

Sequencing of miRNAs in conjunction with public databases was employed to identify miRNAs within the imprinted *Dlk1*-*Gtl2* region on chromosome 18, suggesting their potential roles as epigenetic regulators of fur traits. Small RNAs located at the *Dlk1*-*Gtl2* locus were identified as having the potential to systematically regulate the PI3K-AKT signaling pathway, thereby indicating the relevance of the *Dlk1*-*Gtl2*/PI3K-AKT axis in the context of fur traits. Selection of parental specific expressed imprinted genes in the process of conserving and exploiting lamb fur traits should be emphasized.

**Supplementary Information:**

The online version contains supplementary material available at 10.1186/s12864-023-09741-3.

## Background

The Tan sheep is a distinctive and advantageous local breed in China, concentrated in Ningxia Yanchi and Zhongwei [1]. The complex landscape and unique ecological environment of the region have contributed to the sheep’s high resistance to adversity and tolerance to rough feeding [2]. The lamb fur is one of the core breed characteristics of Tan sheep [3].Its “Ermao” is particularly famous for its resemblance of a flower spike, and is known as “wheat spike flower”, “turnip silk flower” or “mung bean silk flower ”. It is also known as the “string flower”, which has high ornamental value [3].

Ecological studies have shown that the breeding of Tan sheep is distinctly regional, and that the effect of breeding depends on the ecological characteristics and ecogeographical rules [4]. The genetic characteristics of Tan sheep and the role of genetic laws are influenced by environmental interactions [5]. Thus, Tan sheep are ideal material for studying the interaction between epigenetic and ecological laws of livestock [6]. It is important to understand the contribution of epigenetic inheritance to the core breed characteristics of Tan sheep [7]. The fur of Tan sheep displays stage-specific characteristics, specifically observed in the newborn sheep and absent in adults [8]. As a result, numerous studies have focused on exploring the molecular mechanisms underlying fur traits by conducting comparative analyses between newborn and adult stages. Substantial amounts of valuable data have been generated through techniques such as miRNA sequencing [9], messenger RNA (mRNA) sequencing [10], and whole genome methylation sequencing [7], providing a comprehensive dataset for further investigation and analysis. However, the exact epigenetic regulatory loci for fur traits have not yet been reported.

Given that fur traits are significantly influenced by age and environment, we focused our study on epigenetic analysis and conducted miRNA sequencing of sheep skin at 1 and 24 months of age. To search for genetic patterns, we particularly focused on the chromosome distribution of differentially expressed miRNAs and identified the main miRNA loci. This study provides correlation loci for the fur trait of Tan sheep and Hu sheep, and potential novel molecular targets for their breeding and improvement.

## Results

### Analysis of small RNA data obtained from Tan sheep

**Fig. 1:**
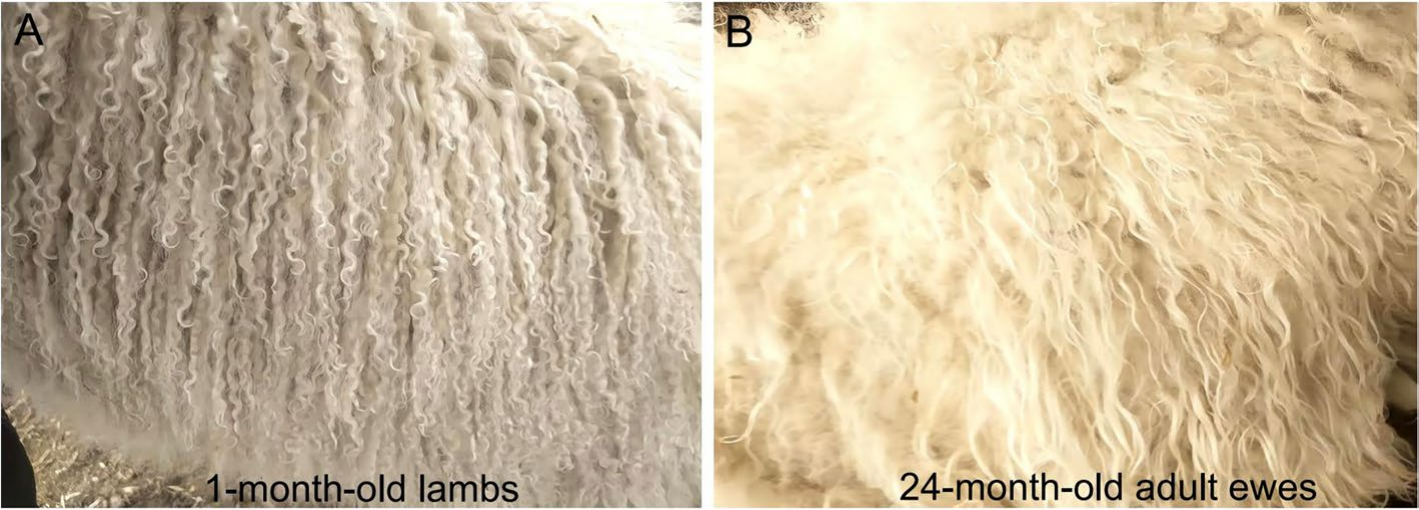
Identifying the fur phenotype of Tan sheep at different periods. **A** The fur phenotype of Tan sheep at newborn period (1-month-old lambs). **B** The fur phenotype of Tan sheep in adulthood (24-month-old ewes)

**Fig. 2:**
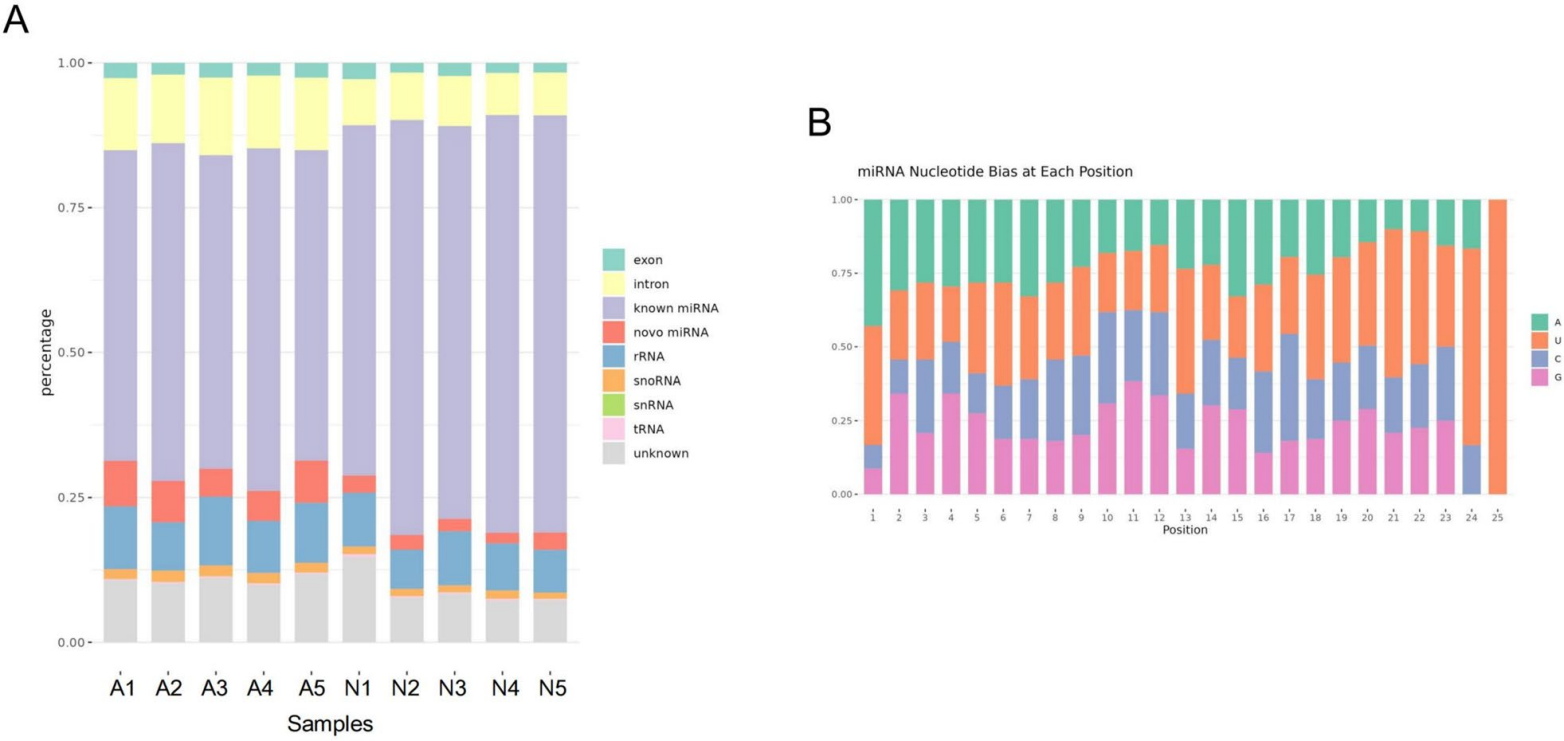
Identification and characterization of miRNAs. **A** Classified identification of all small RNA in all samples. **B** First base preference analysis of known miRNA in all samples

Skin tissue from lambs at the newborn and adult stages was used for small RNA sequencing (Fig. 1), with a total of 18,451,533 to 27,654,766 raw reads were generated (Additional file 1: Table S1). Clean reads numbering of 16,125,840 to 24,463,699 were obtained by filtering and used for subsequent miRNA expression analysis (Additional file 1: Table S1).The lengths of small RNAs were visualized in a distribution map, revealing a notable concentration of miRNAs within the range of 21 to 22 nt in length (Additional file 2: Fig. S1). The distribution analysis of miRNA reads across chromosomes revealed widespread distribution across all chromosomes, with chromosomes 1, 2, and 3 displaying the highest concentrations. (Additional file 3: Fig. S2). From them, up to 149 known miRNAs, 106 precursor miRNAs (Additional file 7: Table S5), 181 new miRNAs (Additional file 8: Table S6), tRNAs (Fig. 2A), ribosomal RNAs (rRNAs) (Fig. 2A), etc. were identified by sequence alignment and annotation. Base preference analysis showed that the first base of the 20 to 24 nt miRNAs is predominantly U, which is consistent with the general profile of miRNAs (Fig. 2B).

### Differential expression analysis of miRNAs points to sheep chromosome 18

**Fig. 3:**
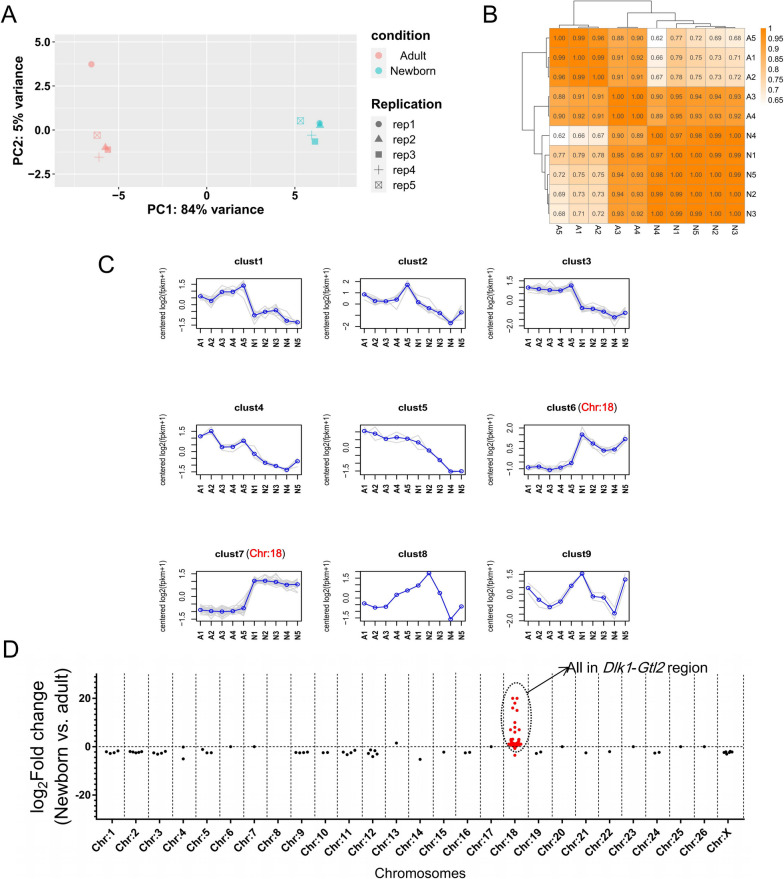
Analysis of miRNA expression between newborn and adult groups. **A** Principal component analysis of miRNA expression between newborn and adult groups. The horizontal coordinate is the first principal component and the vertical coordinate is the second principal component. Different shapes in the figure indicate different samples, and different colors indicate different groupings. **B** Correlation cluster analysis between samples. The higher the correlation between individuals in fig. 3B, the closer the background color. **C** Grouped clustering analysis based on miRNA expression patterns. The gray line shows the expression pattern of miRNAs in each cluster, and the blue line indicates the average of expression of all genes in the cluster in the samples. **D** The distribution of all identified miRNAs across various chromosomes was quantified based on their fold change in expression levels

The results of the principal component analysis (PCA) showed that the newborn and adult groups were completely separated, indicating that there were considerable inter-group differences in miRNA expression profiles between the newborn and adult groups (Fig. 3A). Correlation analysis of miRNA expression levels among samples showed a higher correlation among samples in the newborn group compared to that in the adult group, echoing the PCA results that showed tighter aggregation between samples in the newborn group compared to that in the adult group (Fig. 3B). miRNA expression analysis showed that a total of 149 known miRNAs were detected (Additional file 4: Table S2), of which a total of 80 miRNAs were significantly differentially expressed in the newborn and adult groups (corrected *P*-value < 0.05, |log_2 _fold change| > 1), with 32 up-regulated miRNAs and 48 down-regulated miRNAs in the newborn group ( Additional file 4: Table S2). Furthermore, hierarchical cluster analysis (representing similar gene expression trends within each cluster) remarkably showed that cluster 6 and cluster 7 exhibited considerable differences between the newborn and adult groups (Additional file 5: Table S3). Intriguingly, the differentially expressed miRNAs within both clusters were mapped to sheep chromosome 18. (Fig. 3C). In addition, among all the known miRNAs detected, the majority of those with high expression levels in the newborn group were located on chromosome 18 (Fig. 3D).

### All up-regulated differentially expressed miRNAs are located in the imprinted *Dlk1-Gtl2 *locus

**Fig. 4:**
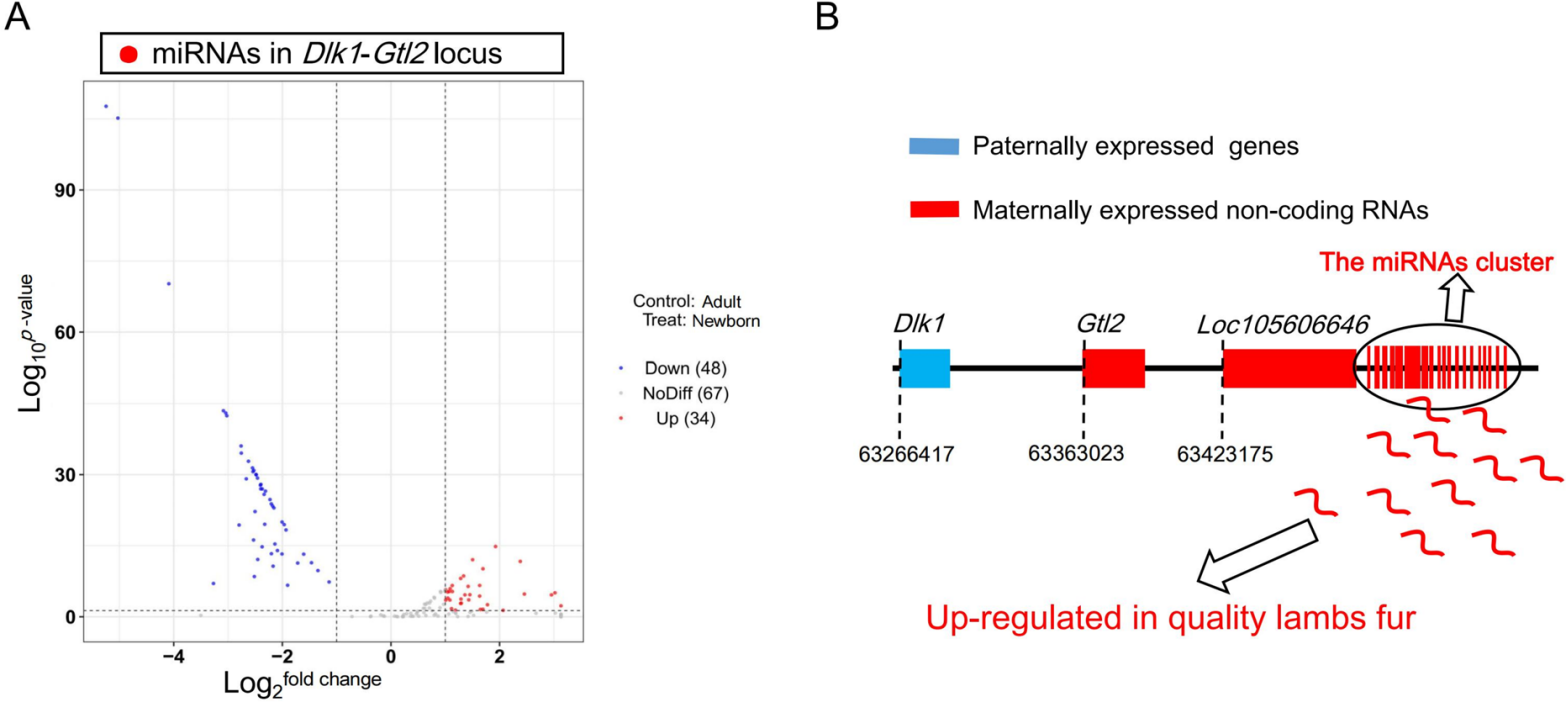
Analysis of differentially expressed miRNAs between newborn and adult groups. **A** The volcano plot of differentially expressed miRNAs. **B** Schematic representation of the imprinted gene Dlk1-Gtl2 locus

By searching the location of each differentially expressed miRNA on the sheep genome, it was found that all differentially expressed miRNAs up-regulated in the newborn group were located at the imprinted gene *Dlk1-Gtl2* locus (Fig. 4A), which encodes maternally expressed long non-coding RNAs, such as *Gtl2*, *Loc105606646*, and a series of miRNAs (Fig. 4B). The 32 differentially expressed miRNAs were up-regulated in the newborn group accounted for 31.7% of the total miRNAs at this locus (Additional file 9: Table S7). However, of the 48 down-regulated miRNAs in the newborn group, none were located at the *Dlk1-Gtl2* locus, but were scattered throughout the genomic chromosomes (Additional file 9: Table S7). This suggests that a number of miRNAs from the *Dlk1-Gtl2 locus* up-regulated in newborn lambs are associated with fur traits.

### The differentially expressed miRNAs associated with fur traits identified in the Ensembl database were also concentrated at the *Dlk1*-*Gtl2 *region

**Fig. 5:**
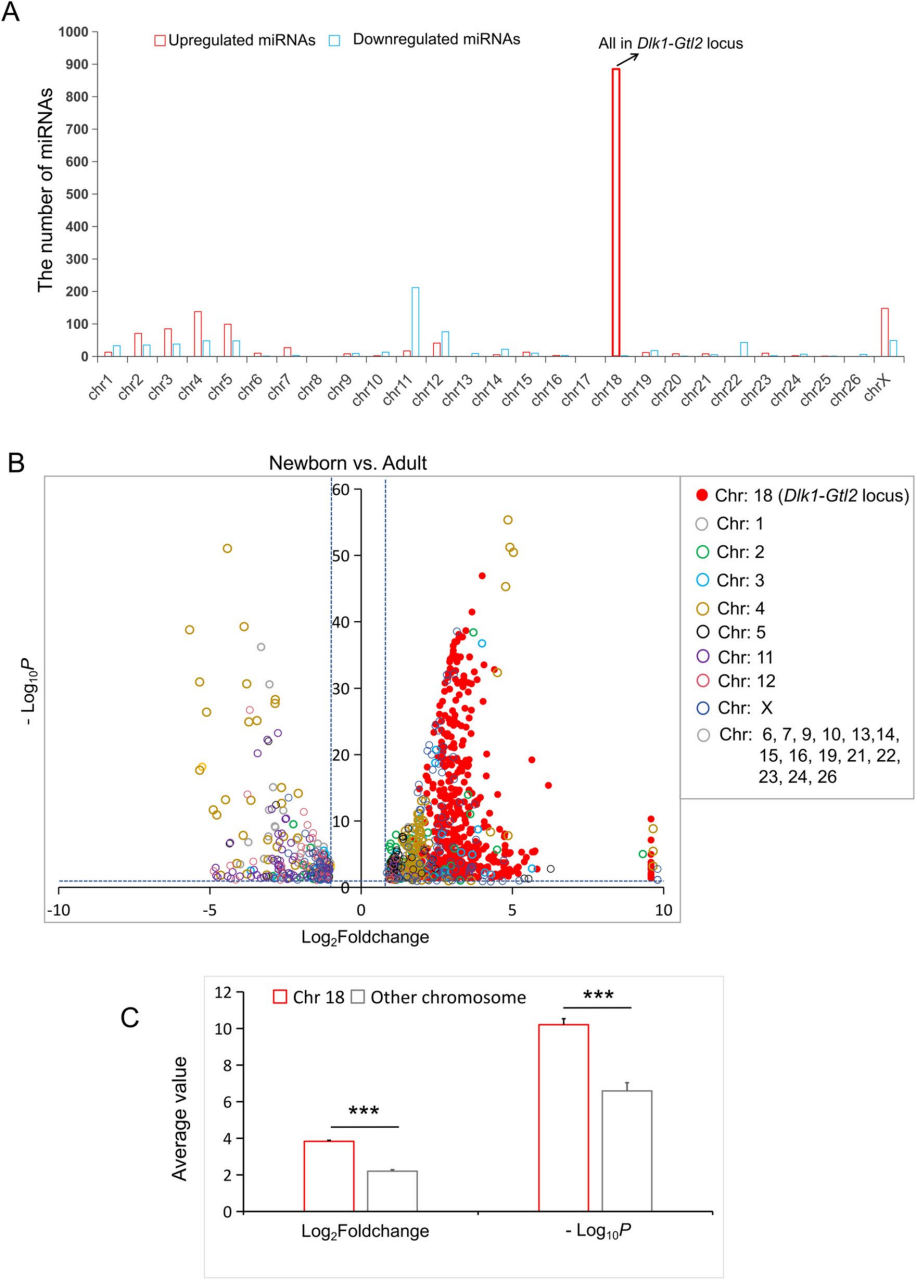
Reanalysis of the miRNA raw data using the Ensembl database. **A** The distribution pattern of differentially expressed miRNAs on chromosomes (Ensembl database). **B** Volcano plots of differentially expressed miRNAs on different chromosomes obtained using the Ensembl database. **C** Comparative analysis of the log_2 _fold change and -log_10_*P*-values of differentially expressed miRNAs on chromosome 18 and other chromosomes, ***，*P *<0.001

In order to identify a broader set of differentially expressed miRNAs, we conducted a comparison of the obtained clean reads with the Ensembl database [11, 12], resulting in the identification of 1,606 up-regulated miRNAs and 693 down-regulated miRNAs (Additional file 13: Table S10). Among these, a significant subset of 885 up-regulated miRNAs (55.1% of 1,606) were found in the the *Dlk1*-*Gtl2* imprinted region (Fig. 5A).

From a chromosomal perspective, it was observed that among all differentially expressed miRNAs, only the up-regulated miRNAs on chromosome 18 exhibited significant positional specificity, the *Dlk1*-*Gtl2* locus, and they were all highly expressed in the high-quality fur group (Fig. 5B and additional file 12: Table S9). Furthermore, the log_2 _fold change and -log_10_corrected *P*-value of the up-regulated miRNAs at the *Dlk1*-*Gtl2* locus were both higher than those of other differentially expressed miRNAs, *P* < 0.001 (Fig. 5C).

### PI3K-AKT is a potential co-regulatory signaling pathway for miRNAs in the *Dlk1*-*Gtl2 *region

**Fig. 6:**
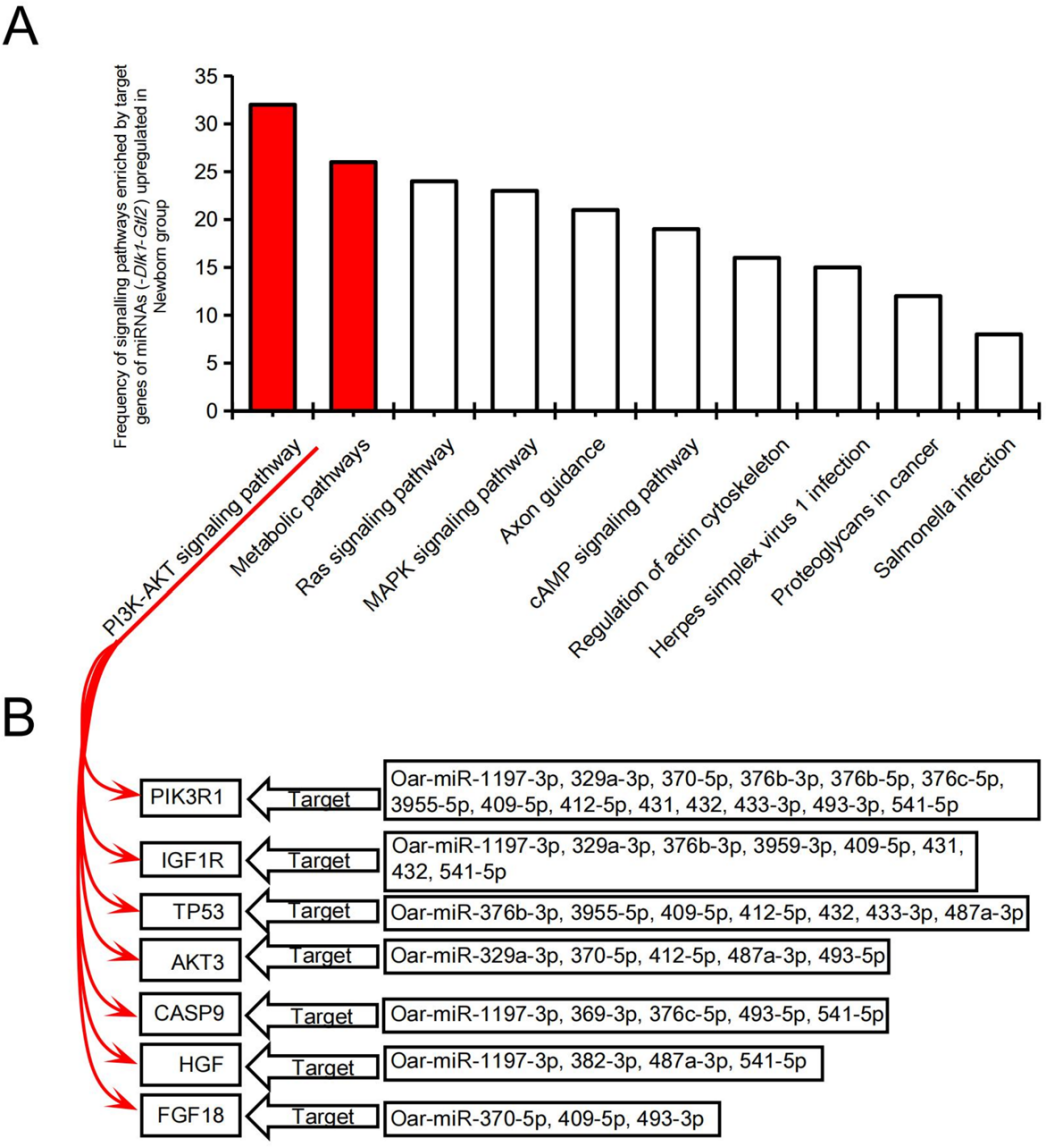
Functional enrichment analysis of target genes for candidate miRNAs in *Dlk1-Gtl2 *locus. **A** Pooled analysis of the signaling pathways enriched to the target genes of all up-regulated miRNAs in newborn groups. **B** Marker genes in the PI3K-AKT signaling pathway and genes associated with hair follicle development

 To explore the potential molecular mechanisms regulating fur traits in newborns, miRanda software [13] was used to predict the potential target genes for each differentially expressed miRNA (Additional file 6: Table S4). We performed a functional enrichment analysis of the target genes for each of the upregulated miRNAs in the newborn group, ranked each of the obtained signaling pathways in terms of *P*-value and the number of enriched genes, and took the top ten signaling pathways in each group for subsequent analysis. The frequency of the top ten signaling pathways in the 32 groups was further counted and then ranked (Fig. 6A). The PI3K-AKT signaling pathway was in first place and the key genes on this pathway have the potential to regulate hair follicle development such as *TP53* [14, 15], *HGF* [16–18] and *FGF18* [19–21] (Fig. 6B).

Functional enrichment analysis of signaling pathways by miRNA target genes showed that metabolic pathways did not occur as frequently as PI3K-AKT signaling pathway (Fig. 6A), however, among the miRNA target genes, more genes were enriched to metabolic pathways than PI3K-AKT signaling pathway (Additional file 10: Fig. S3).

### Functional enrichment analysis of down-regulated small RNAs

**Fig. 7:**
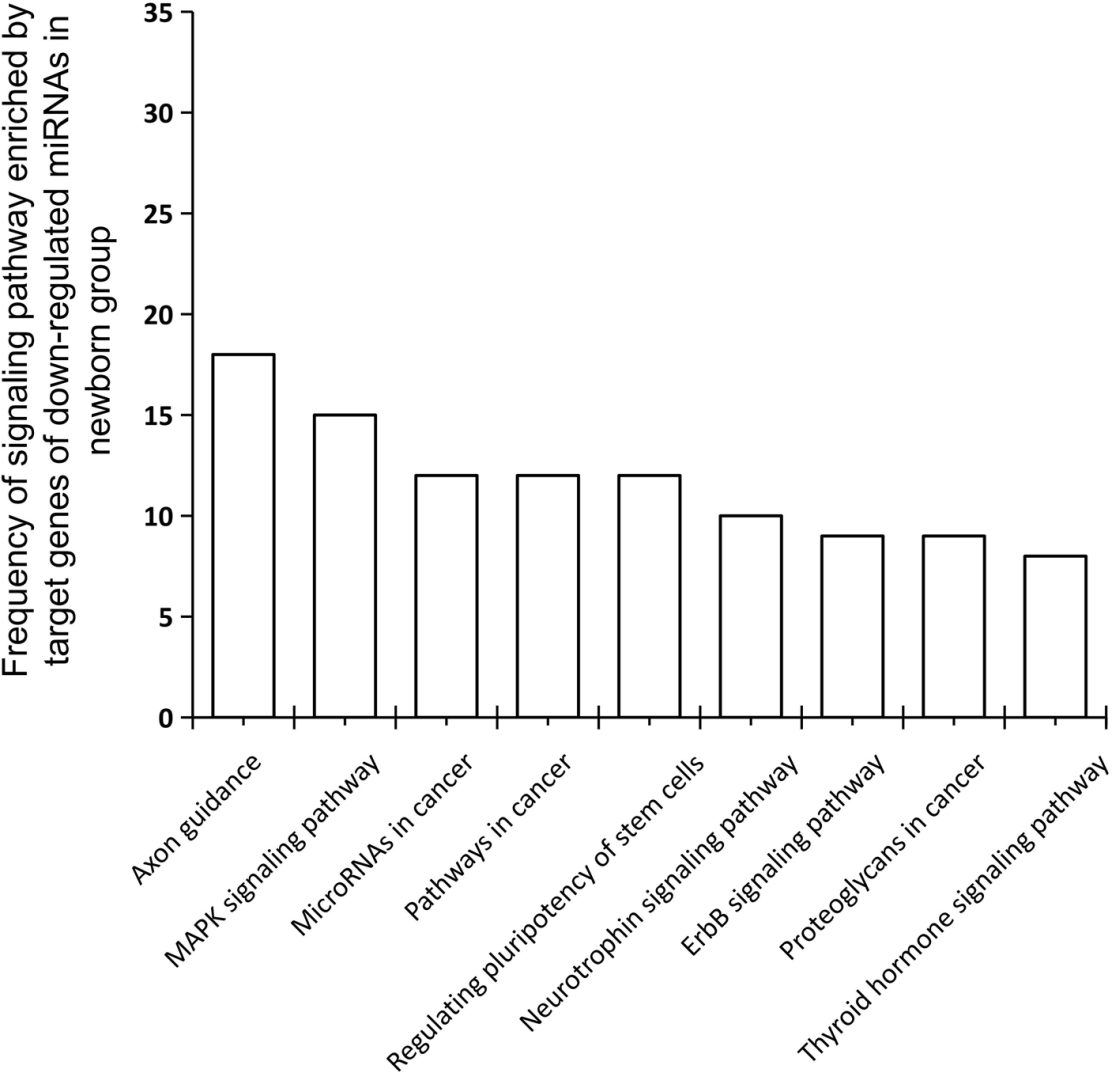
Functional enrichment analysis of target genes for down-regulated miRNAs in newborn group

 All the target genes of down-regulated miRNAs in the 1-month-old group were predicted, and functional enrichment analysis was performed for target genes of each down-regulated miRNAs. The results showed that the highest frequency of each signaling pathway enriched to the target genes of down-regulated miRNAs was 18 times, which accounted for 37.5% of all down-regulated miRNAs. In contrast, the highest frequency of enriched signaling pathways for the target genes with up-regulated miRNAs was 32 times, which accounted for 94.1% of all up-regulated miRNAs (Fig. 7).

### The validation of differentially expressed miRNAs using reverse transcription quantitative real-time polymerase chain reaction (RT-qPCR)

**Fig. 8:**
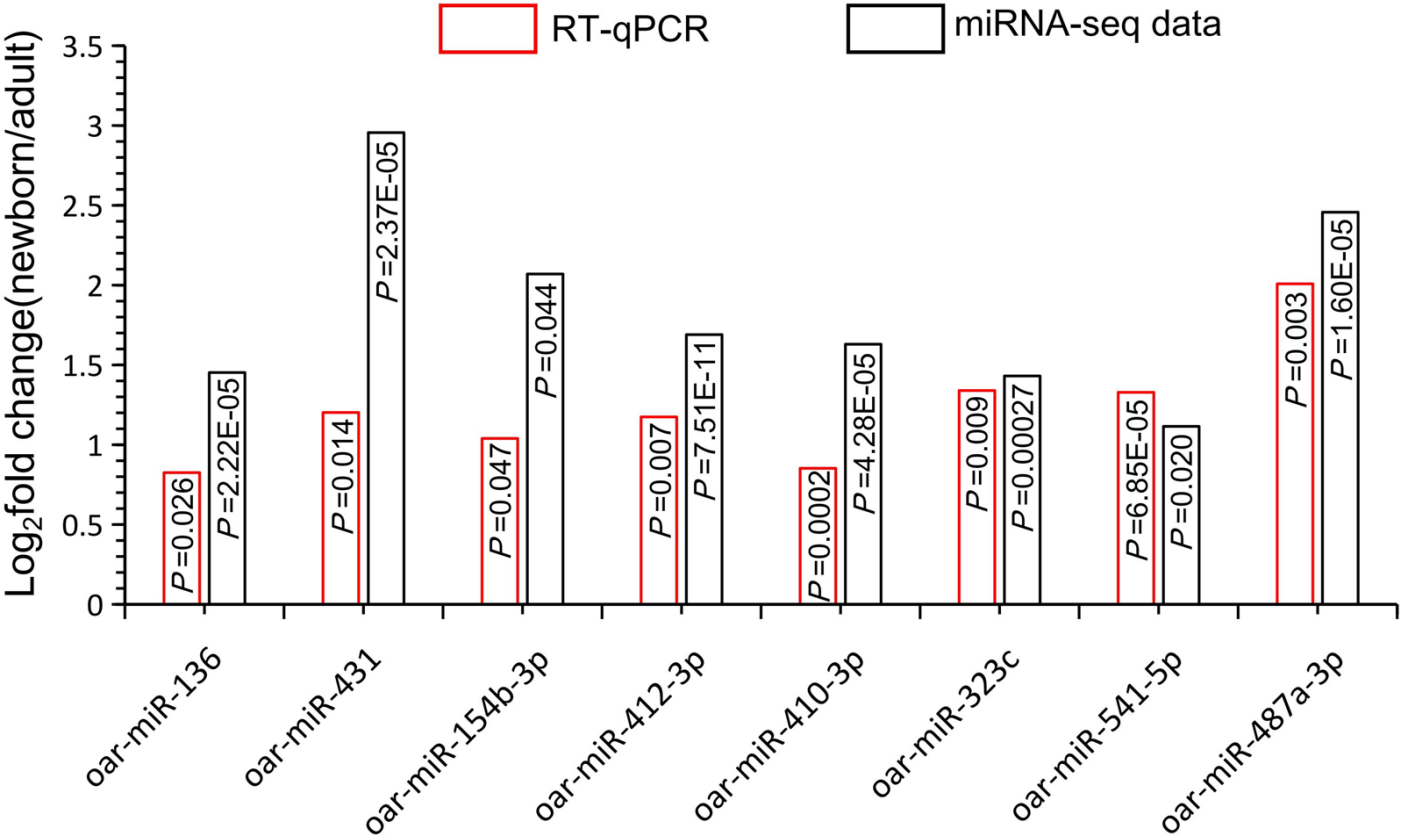
Relative expression of differentially expressed miRNAs by RT-qPCR in newborn and adult group, *n *= 3, *P *< 0.05

 Eight miRNAs located on chromosome 18 were selected for RT-qPCR validation to confirm the accuracy of miRNA sequencing results. The results showed that all eight miRNAs were significantly upregulated in the skin tissues of newborn lambs, regardless of whether they were quantified by RT-qPCR or miRNA sequencing techniques (Fig. 8).

## Discussion

The *Dlk1*-*Gtl2* locus is an important imprinted gene region in mammals, it contains the paternally expressed imprinted genes such as *Dlk1* and *Rtl1*, as well as non-coding long non-coding RNAs and a number of small RNAs [22]. This locus is essential for normal mammalian embryos development [23]. It has been shown that silencing of the imprinted gene *Rtl1* is a major cause of early developmental failure in cloned mammalian embryos after implantation [24]. An A to G mutation in this region causes the callipyge phenotype (CLPG), altering skeletal muscle development [25]. More importantly, small RNAs in this region systematically inhibit the PI3K-AKT-mTOR signaling in hematopoietic stem cells, which in turn restricts their mitochondrial metabolism [26]. This is consistent with our functional analysis.

Tan sheep are not the only Chinese strain used for studying fur traits, Hu lambs are also well known for their fur, and their watery wool spikes are favored by consumers globally [27]. We studied both newborn and adult samples, which contained two variables: fur differences and age differences. To reduce the number of experimental variables, we performed a reported study, in which Hu lambs with high-quality lamb fur were selected as the case group and those with low-quality fur as controls for miRNA sequencing [27]. Among the differentially expressed miRNAs in this study, a total of 14 up-regulated known miRNAs and four down-regulated miRNAs in lambs with high-quality fur (Additional file 11: Table S8). Of the top eight up-regulated miRNAs in high-quality lamb fur group, seven were in the imprinted *Dlk1-Gtl2* locus (Additional file 11: Table S8). This further suggests that these miRNAs may be the potential regulatory locus for fur traits.

Our miRNA sequencing revealed that miRNAs in the *Dlk1-Gtl2* region are associated with fur traits, suggesting that the molecular mechanisms regulating fur may be very complex. In fact, the vast majority of these miRNAs regulate the PI3K-AKT signaling pathway, demonstrated in humans and mice [26, 28, 29]. Given that miRNAs and their targets are highly evolutionarily conserved [30–33], we hypothesize that miRNAs from the *Dlk1*-*Gtl2* region regulate lamb fur development through PI3K-AKT signaling.

To better assess whether PI3K-AKT affects lamb fur quality we note that fur quality is a function of the wool fibers, with a pleasing wavy appearance; in turn, the quality of wool fibers is determined by the developmental states of hair follicles [34]. It has been shown that PI3K signaling is required for hair follicle development [30, 35–37] supporting a role for PI3K-AKT signaling in regulating fur quality. In addition, Chinese livestock ecologists have systematically observed and studied Tan sheep from an ecological perspective [38] and found that they exhibit high-quality fur traits under poor nutritional conditions and low-quality fur traits under better nutritional conditions [38]. Given that IGF1 and IGF2 levels are gold standards for identifying malnutrition [39–41], and that they are major upstream regulators of the PI3K-AKT signaling pathway [42–45]. we hypothesise that when Tan sheep are exposed to nutritional deprivation, IGF1 and IGF2 levels are decreased, which in turn inhibits PI3K-AKT signaling, consistent with the effect achieved by miRNAs in the *Dlk1-Gtl2* region on the general inhibition of PI3K-AKT signaling observed in this study. This suggests that Tan sheep fur characteristics are subject to endogenous epigenetic regulation, which is a breed characteristic. Furthermore, to increase fur quality, we cannot ignore the role of specific ecological factors including nutrition in regulating PI3K-AKT signaling.

## Conclusions

In this study, miRNA sequencing showed that the expression of numerous small RNAs within the *Dlk1-Gtl2* region is associated with quality lamb skin traits, and the potential involvement of *Dlk1*-*Gtl2*/PI3K-AKT axis in the regulation of lamb fur quality. These findings have important implications in wool production.

## Methods

### Sample collection and total RNA extraction

The entire animal experimentation process strictly adhered to the China Guide for the Care and Use of Laboratory Animals. All experimental operations were approved by the Animal Experimentation Ethics Committee of China Agricultural University (license number: SKLAB-2012-04-07). Ten female Chinese Tan sheep (five of which were 30-day-old lambs, referred to as the newborn group, and the remaining five were 24-month-old adult ewes, referred to as the adult group) were used. The traits of fur quality and age share a significant association. It is especially noteworthy that fur quality exhibits heightened superiority during the newborn stage, while demonstrating a gradual decline in quality during the adult stage. The 10 Tan sheep belonged to the Yanchi County breeding farm in Wuzhong city (located in Ningxia Hui Autonomous Region, China). Through dedicated staff, it was ensured that these 10 sheep were reared under the same conditions. The wool phenotypes of these 10 Tan sheep were similar within the group and differed significantly between groups (Fig. 1). We sampled these 10 Tan sheep at the same time and place, collected skin tissue and immediately froze it in liquid nitrogen. Total RNA was extracted using TRIzol reagent (Invitrogen, CA, USA). Total RNA was extracted according to the instructions for this reagent. RNA concentration and purity were measured using a NanoDrop 2000 (Thermo Scientific, Waltham, Massachusetts, USA), and integrity was measured using a Agilent 2100 Bioanalyzer.(Agilent Technologies Inc, California, USA). The RNA samples were used for subsequent miRNA sequencing. Permission was obtained from the owners of the farm and the animals.

### Library preparation for sequencing

The small RNA libraries were generated using the NEBNext® Multiplex Small RNA Library Prep Set for Illumina® (NEB, Ipswich, MA, USA), with 2 µg of total RNA per sample used to generate miRNA libraries. Specifically, the 3’ and 5’ end adaptor were directly and specifically linked respectively to the 3’ and 5’ ends of miRNA, siRNA and piRNA. The first strand of cDNA was then synthesized using M-MuLV reverse transcriptase and PCR amplified. DNA fragments of 140–160 bp were purified in the eluate buffer. The cDNA libraries were evaluated on an Agilent Bioanalyzer 2100 system using a DNA high sensitivity chip and then the libraries were sequenced on an Illumina Hiseq 2500 platform. The image files were transformed by the software of the sequencing platform to generate Raw Data of FASTQ raw downstream data. When the average sequencing quality of the bases in the window was below 20, the part starting from the top of the window was truncated and discarded. The total number of clean reads with sequence length between 18 nt and 36 nt was counted. Identical sequences within a single sample were de-duplicated and counted for sequence abundance, and were called unique reads for subsequent analyses.

### Reference genome alignment

The sequencing data were aligned to the genome and genome alignment analysis was performed using miRDeep2 software, where the mapper.pl program calls Bowtie v2.5.1 to perform alignment between the unique reads and reference genome sequences [46]. Reads that were mapped to reference sequences were aligned to sequences in miRBase [47]. The reference genome used in this analysis is GCF_016772045.1 on the National Center ofr Biotechnology Information (NCBI) website. Small RNA were mapped to the Rfam database and RepeatMasker, thereby removing repetitive sequences and tags formed by mRNA. Mireap (https://sourceforge.net/projects/mireap/) and miRDeep2 software [46] were used to search for novel miRNAs using parameters such as minimum free energy (the threshold for MFE is -18 kcal/mol), secondary structure of Dicer cleavage sites and unnamed small RNA tags, first nucleotide bias for different lengths of miRNAs and miRNA nucleotide bias at each position. According to the priority rule, known miRNAs were first mapped and annotated with small RNAs, followed by rRNA, tRNA, snRNA and snoRNA. The miRDeep2 parameters were the default: -c, -e fastq, -h, -j, -k, -l 18, -m, -p, -s, -t, -d. In addition, we also aligned the sequencing data with the Ensembl database. Blastn was used to align sequencing reads with the precursor sequences of miRNAs in the sheep genome (ovis_aries_rambouillet, The release-110 version of the reference genome) in the Ensembl database. Then the matched readings were used as a miRNA mature library and the corresponding reads of mature miRNAs in different samples.

### Differential expression of miRNA

In this study, Bowtie2 (v2.5.1) software was used to count miRNA reads based on the number of sequences matched to the mature sheep miRNAs [48]. Benjamini - Hochberg method was used for corrected *P*-values. The two parameters (fold change and corrected *P*-values) were used to assess significant differences in the expression levels of miRNAs between these two groups. Corrected *P*-values < 0.05 and |log_2 _foldchange| > 1 were considered as indicative for miRNAs with significant differential expression. As there were five biological replicates in the newborn and adult groups, the DESeq2 (v1.39.0) R package was used for differential expression analysis between these two groups. The read counts were used in “DESeq” package. In addition, volcano plots were used to screen for differentially expressed miRNAs.

### Target gene prediction and functional enrichment analysis of miRNAs

The 3 ’UTR sequence of the mRNA of this species was considered to be the target sequence, and MiRanda (v3.3a) was used to perform target genes prediction for the differentially expressed miRNA sequence. [13]. These genes were then subject to functional enrichment analysis via David (https://david.ncifcrf.gov/tools.jsp) [49, 50], The target genes of each miRNA were found to be enriched across various signaling pathways, which were subsequently ranked based on their frequency of occurrence in different miRNA target pathways. The magnitude of this frequency directly corresponds to the number of miRNAs involved in regulating each respective signaling pathway. Results from miRanda were screened based on Max Score > 150 and Max Energy<-20. Additionally, TargetScan (https://www.targetscan.org/vert_80/) was utilized to predict the target genes of up-regulated miRNAs. The predicted results were then intersected with those from miRanda conditional screening to plot an interaction network between miRNAs and target genes.

### RT-qPCR

The manufacturer’s protocol was used to extract total RNA from skin tissues of 1-month-old (*n* = 3) and 24-month-old (*n* = 3) Tan sheep using TRIzol reagent (15,596,026, Invitrogen, Carlsbad, CA, USA). These six sheep skin samples were the remaining samples used for small RNA sequencing (A1 to A3 and N1 to N3). Primers for *Oar-miR-136, Oar-miR-431, Oar-miR-154b-3p, Oar-miR-412-3p, Oar-miR-410-3p, Oar-miR-323c, Oar-miR-541-5p, Oar-miR-487a-3p*, and *U6* were obtained from Guangdong Ruibo Company (Ribobio, Guangzhou, China), and reverse transcription was performed using the TaqManTM MicroRNA Reverse Transcription Kit (Applied Biosystems by Thermo Fisher Scientific, USA), following the manufacturer’s instructions. SYBR Green qPCR Mix kit (FP205, TIANGEN, Beijing, China) was used for RT-qPCR, which was performed on a BioRad CF×96 (CFX96, BIO-RAD, Hercules, CA, USA) quantitative instrument. The reaction conditions included pre-denaturation at 95 °C for 30 s, denaturation at 95℃ for 10 s, annealing at 59℃ for 30 s, and extension at 72℃ for 30 s for 40 cycles. Each sample was run in triplicate, with *U6* serving as the internal control. The relative expression levels between groups were calculated using the 2^−△△CT^ method [51]. The results were imported into R for difference analysis. The Sapiro Wilk method was used to test whether the data conforms to a normal distribution (*P* > 0.05 means a normal distribution). If the data conformed to a normal distribution, a t-test was used, with the significance threshold *P* < 0.05.

### Statistical analysis

The utilization of DESeq software relies on adhering to a negative binomial distribution, while analysis of variance *p*-values was performed using the nbinomTest function within the DESeq package. The software estimates discrete values and normalizes the read count data.

### Supplementary Information

**Additional file 1: Table S1.** The raw and clean datas of each samples

**Additional file 2: Figure S1. **Length distribution of total small RNA fragments

**Additional file 3: Figure S2. **Genome-wide distribution of small RNAs

**Additional file 4: Table S2.** The list of differentially expressed miRNAs between newborn and adult group

**Additional file 5: Table S3.** Cluster analysis of differentially expressed miRNA groups.

**Additional file 6: Table S4.** The list of predicted differentially expressed miRNA target genes 

**Additional file 7: Table S5. **Statistics on the annotation of known miRNAs

**Additional file 8: Table S6.** Statistics of annotation of new miRNAs

**Additional file 9: Table S7.** Differentially expressed miRNAs between Newborn and Adult group

**Additional file 10: Figure S3.** Interacting analysis was conducted to investigate the relationships between a subset of miRNAs within the *Dlk1-Gtl2* region and their target genes. Triangle symbols represent miRNAs, circle symbols represent target genes

**Additional file 11: Table S8.** Differentially expressed miRNAs between good and poor quality fur groups

**Additional file 12: Table S9.** Analysis of differentially expressed miRNAs in skin tissues of newborn and adult Tan sheep using Ensembl database

**Additional file 13: Table S10. **The statistical analysis of miRNAs obtained using the Ensembl database.

## Data Availability

The miRNA-seq data reported in this study have been deposited in the National Center for Biotechnology Information (NCBI) database with the accession numbers PRJNA967624 (https://www.ncbi.nlm.nih.gov/sra/?term=PRJNA967624).

## Abbreviations

PCA: Principal component analysis
RT-qPCR: Real-time quantitative polymerase chain reaction
