# Supplementary figures and images for: Imprinted Dlk1-Gtl2 cluster miRNAs are potential epigenetic regulators of lamb fur quality
Source: BMC Genomics. 2023 Oct 23;24:632. doi: 10.1186/s12864-023-09741-3 (PMC10594899; doi:10.1186/s12864-023-09741-3)

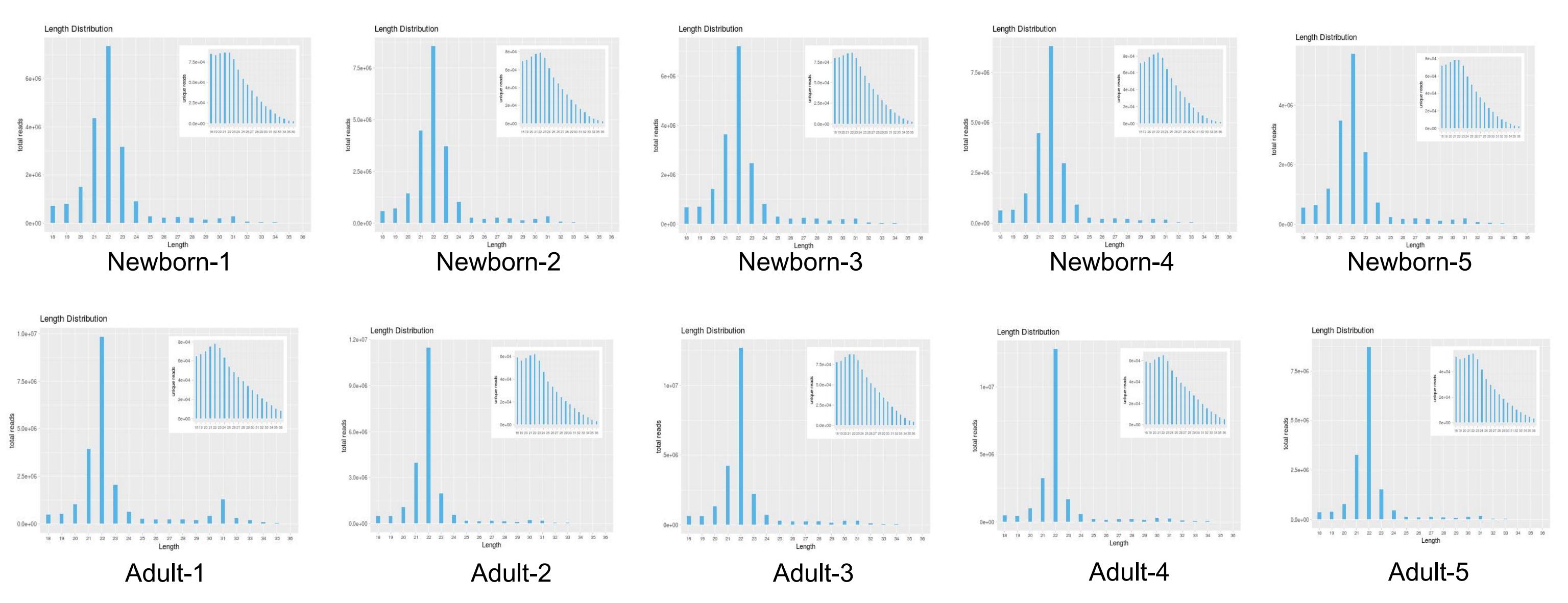


**Additional file 2: Figure S1.** Length distribution of total small RNA fragments.

Supplement: Supplementary file 2 — Additional file 2: Figure S1. Length distribution of total small RNA fragments [file 12864_2023_9741_MOESM2_ESM.docx]

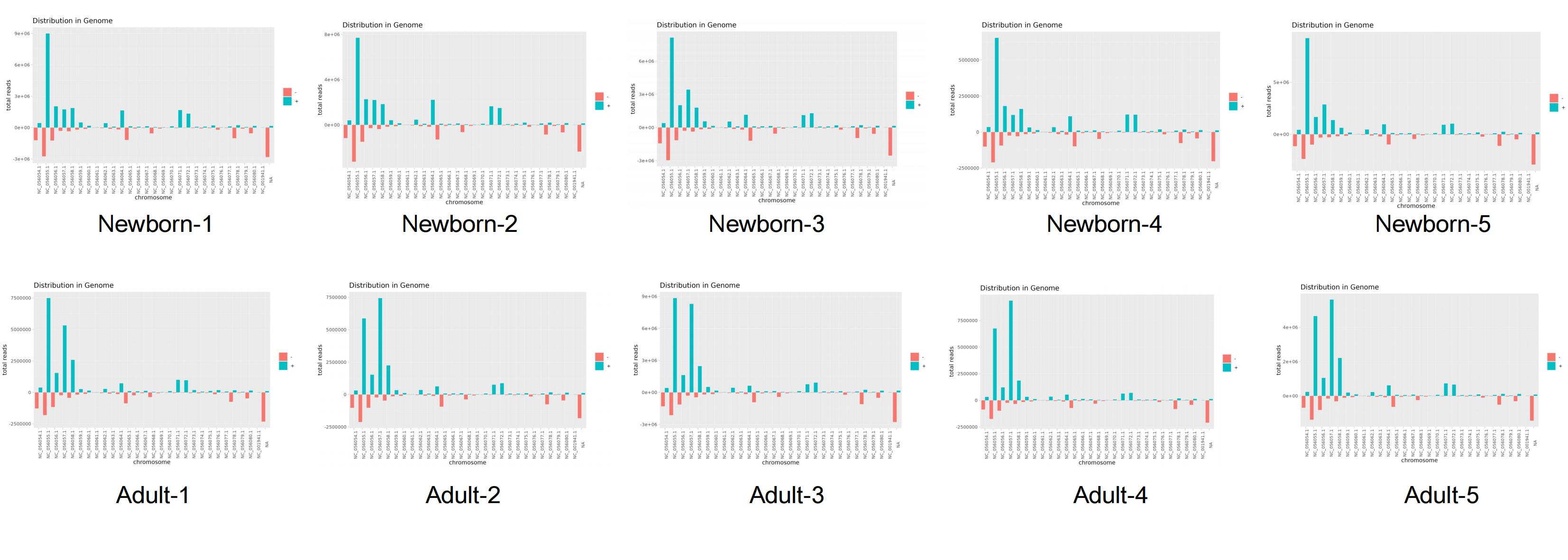


**Additional file 3: Figure S2.** Genome-wide distribution of small RNAs.

Supplement: Supplementary file 3 — Additional file 3: Figure S2. Genome-wide distribution of small RNAs [file 12864_2023_9741_MOESM3_ESM.docx]
