# Supplementary material for: Imprinted Dlk1-Gtl2 cluster miRNAs are potential epigenetic regulators of lamb fur quality
Source: BMC Genomics. 2023 Oct 23;24:632. doi: 10.1186/s12864-023-09741-3 (PMC10594899; doi:10.1186/s12864-023-09741-3)
Supplement: Supplementary file 10 — Additional file 10: Figure S3. Interacting analysis was conducted to investigate the relationships between a subset of miRNAs within the Dlk1-Gtl2 region and their target genes. Triangle symbols represent miRNAs, circle symbols represent target genes [file 12864_2023_9741_MOESM10_ESM.docx]

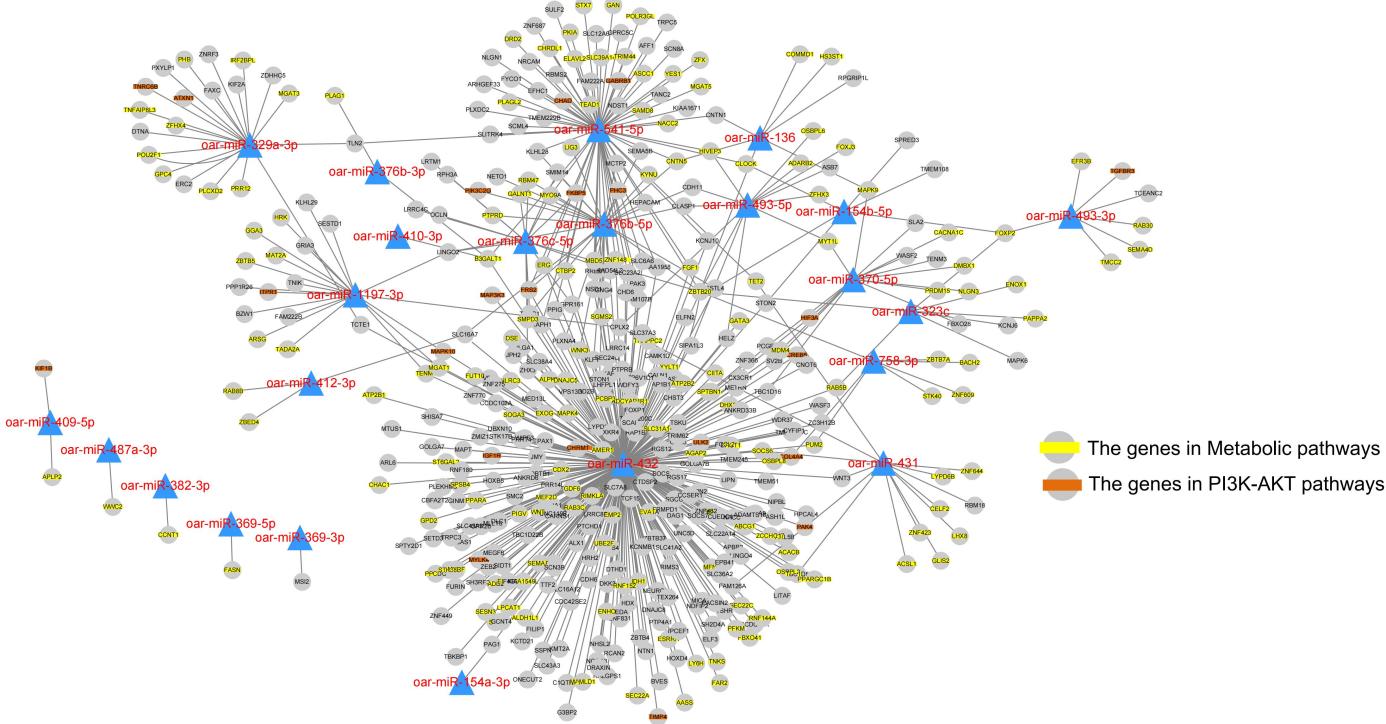


**Additional file 10: Figure S3.** Interacting analysis was conducted to investigate the relationships between a subset of miRNAs within the *Dlk1-Gtl2* region and their target genes. Triangle symbols represent miRNAs, circle symbols represent target genes.
